# Metformin increases pathological responses to rectal cancers with neoadjuvant chemoradiotherapy: a systematic review and meta-analysis

**DOI:** 10.1186/s12957-023-03087-6

**Published:** 2023-07-26

**Authors:** I-Li Lai, Jeng-Fu You, Wen-Sy Tsai, Yu-Jen Hsu, Yih-Jong Chern, Ming-Ying Wu

**Affiliations:** 1 Division of Colon and Rectal Surgery, Chang Gung Memorial Hospital, Guei-Shan District, Linkou Branch, No. 5, Fu-Hsing Street, Taoyuan City, Taiwan; 2 Graduate Institute of Clinical Medical Sciences, College of Medicine, Chang Gung University, No. 259, Wenhua 1St Rd, Guei-Shan District, Taoyuan City, Taiwan; 3 Department of SurgeryTen-Chen Medical GroupZhongli Dist., Zhong-Li Metropolitan Hospital, Yanping Rd, No. 155, Taoyuan City, Taiwan; 4 Department of Dermatology, Chang Gung Memorial Hospital, Guei-Shan District, Linkou Branch, No. 5, Fu-Hsing Street, Taoyuan City, Taiwan; 5 Institute of Epidemiology and Preventive Medicine, Zhongzheng Dist., National Taiwan University, Xuzhou Rd, No. 17, Taipei City, Taiwan

**Keywords:** Rectal cancer, Neoadjuvant chemoradiotherapy, Metformin, Pathological response, Diabetes mellitus, Meta-analysis

## Abstract

**Background:**

To summarize the chemo-radio effect of metformin in rectal cancers with neoadjuvant chemoradiotherapy on pathological response, tumor regression grade (TRG), and T/N downstaging.

**Methods:**

PubMed, MEDLINE, Embase, and Cochrane Database of collected reviews were searched up to June 30, 2022. This study conducted systematic review and meta-analysis based on the Preferred Reporting Items for Systematic Reviews and Meta-Analyses (PRISMA) sheet. Odds ratios (ORs) and confidence intervals (CIs) which calculated by random-effects models were displayed in forest plots. Newcastle–Ottawa scale was used to assess the risk of bias of the observational cohort studies.

**Results:**

This systematic review and meta-analysis comprised eight cohorts out of seven studies, with 2294 patients in total. We performed two-way comparison for metformin in diabetic patients vs (1) non-metformin drugs in diabetic patients and (2) nondiabetic patients. In diabetes patient studies, the metformin group had a significantly increased pathological response on TRG (*OR*: 3.28, *CI*: 2.01–5.35, *I*^2^ = 0%, *p* < 0.001) and T downstaging (*OR*: 2.14, *CI*: 1.24–3.67, *I*^2^ = 14%, *p* = 0.006) in comparison with a non-metformin group. When compared with nondiabetic patients, the pathological response on TRG (*OR*: 2.67, *CI*: 1.65–4.32, *I*^2^ = 43%, *p* < 0.001) and T downstaging (*OR*: 1.96, *CI*: 1.04–3.71, *I*^2^ = 66%, *p* = 0.04) were also higher in metformin group. The limitation was that no randomized controlled trials were available based on current literature review. Small sample sizes for diabetic metformin or non-metformin users in rectal cancer patients reduced the power of the study.

**Conclusions:**

For patients with rectal cancer and treated with neoadjuvant chemoradiotherapy, metformin administration in diabetic patients increased the pathological response on tumor-regression grade and T downstaging. Further well-designed, high-quality randomized controlled trials are required to reveal the actual effect of metformin.

**Supplementary Information:**

The online version contains supplementary material available at 10.1186/s12957-023-03087-6.

## Introduction

Colorectal cancer (CRC) remains the third most commonly occurring cancer with the second most deaths worldwide [1]. Rectal cancer, accounts for about one-third of all CRCs, is hard to achieve adequate surgical margins and has a higher local recurrence rate [1].

For better local control and survival outcomes, current guidelines suggest rectal cancer at stages 2 or 3 should receive neoadjuvant radiotherapy (RT) or chemoradiotherapy (CRT) followed by total mesorectal excision. Reviewing the post-neoadjuvant radical resection tissues, there is approximate 12 − 20% pathological complete response (pCR) rate [2]. Achieving pCR brings a lower local recurrence rate and a higher disease-free survival rate [2–4].

Metformin, the most commonly prescribed first-line hypoglycemic agent, showed benefits not only in diabetes treatment but also in lowering the risk of developing colorectal adenomas and CRCs [5]. Some basic studies have reported a radiosensitivity effect for metformin and have indicated it may enhance pCR in rectal cancers after neoadjuvant CRT. However, clinical evidence remains scarce and has shown no consensus. Our study included a systematic review with meta-analysis on the effect of metformin on rectal cancers with neoadjuvant CRT.

## Material and methods

The review was synthesized with the Preferred Reporting Items for Systematic Reviews and Meta-Analysis (PRISMA) guidelines [6]. It complied with the PRISMA, and the study design was registered on PROSPERO, CRD42022369841.

### Search strategy

Electronic searches were conducted on MEDLINE (PubMed), Embase, and Cochrane Library databases from inception to June 30, 2022. The search strategy included the following terms: “(colo-)rectal cancer” or “(colo-)rectal neoplasm” or “(colo-)rectal (adeno-)carcinoma” and “radiotherapy” or “chemotherapy” or “chemoradiotherapy” or “neoadjuvant” or “preoperative” and “metformin” or “biguanide” or “oral hypoglycemic agent.” The details of the search strategy can be found in Supplementary file 1.

### Inclusion and exclusion criteria

The inclusion criteria were the following: (1) case–control studies in which the intervention treatments were neoadjuvant CRT with or without metformin in CRC patients and (2) cohort studies that studied the response of CRT in CRC patients following metformin, non-metformin hypoglycemia agents, and/or insulin.

The exclusion criteria were the following: (1) case reports, reviews, letters to the editor, or discussions; (2) studies include colon or colorectal cancers, but rectal cancers could not be extracted; and (3) preoperative neoadjuvant CRT was not performed, or the effect of response could not be extracted.

### Data extraction

We extracted information on aggregate study-level participant characteristics [i.e., total included patients, age, gender, body mass index, clinical stage of rectal cancers, pretreatment carcinoembryonic antigen (CEA), glycosylated hemoglobin (HbA1c) value, blood sugar value, diagnosis of diabetes mellitus (DM), and metformin treatment details]. As all included patients had rectal cancer undergoing neoadjuvant CRT, the treatment details regarding doses and duration of RT, regimens and doses of chemotherapy, and the following curative surgery were extracted.

The outcomes of interest were responses of CRT. Therefore, we extract available pathological responses (pathological response rate, TRG and T/N downstaging rate) of these patients. In these including studies, pathological response and T/N downstaging were binary data which consists of yes and no. Depending on the system of TRG, this data ranged from 0 to 4. We also converted the TRG data to binary data which consists of good and poor. After the outcome data were extracted as binary data, we performed the meta-analysis, and the pooling effect sizes were odds ratio.

For rectal cancer with neoadjuvant CRT followed by curative resection, pathologists examine the specimen and determine the pathological tumor and node staging following neoadjuvant CRT and TRG to restage. TRG is scored by the proportion of residual tumor cells and fibrosis. The most extensively used TRG systems are AJCC system, Dworak, and Mandard systems [7]. To compare the outcome from different studies, we graded Mandard TRG 1 and 2 and Dworak TRG 4 and 3 as the same as AJCC TRG 0 and 1. According to AJCC system, TRG scored 0 (no remaining viable cancer cells) or 1 (only small clusters or single cancer cells) was regarded as a good response to CRT. Putting together, we compared the odds ratio of pCR, TRG, and T/N downstaging after CRT in two-way comparison for metformin in diabetic patients vs (1) non-metformin drugs in diabetic patients and (2) nondiabetic patients.

If the eligible study did not offer some of the outcome data, then the study would be excluded from synthesis. Two review authors were assigned to independently perform the data extraction. Any disagreement was reviewed and decided by the third involved author, Yu-Jen Fu.

### Quality assessment

As all the included studies were retrospective cohort studies, they were independently assessed using the Newcastle–Ottawa scale (NOS) for the study quality by two authors. The NOS respectively examined the selection, comparability, and ascertainment of study groups regarding exposure and the outcome of interest in cohort studies [8].

### Statistical analysis

In this study, a meta-analysis was conducted to analyze the odds ratio (OR) for binary variables. The results were presented in forest plots, which included 95% confidence intervals (CIs). Heterogeneity measures were performed using RevMan 5.4 version and included *I*^2^, tau, and Cochran Q tests. An *I*^2^ value between 0 and 25% was considered non-significant, while a value between 25 and 60% suggested moderate heterogeneity, and a value over 60% suggested substantial heterogeneity. To perform this meta-analysis, a DerSimonian-Laird random effects model was utilized. We used Begg funnel plot and Egger’s linear regression test to identify publication bias in the literature reviewed. *P*-value < 0.05 was used to indicate the significance.

## Results

### The literature review process

**Fig. 1:**
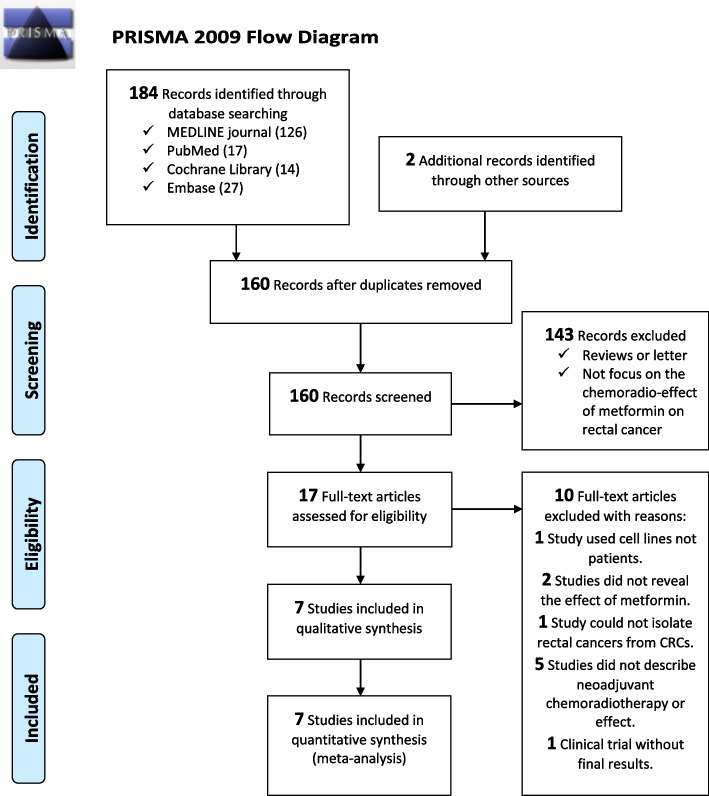
Preferred Reporting Items for Systematic Reviews and Meta-Analyses (PRISMA) sheet

Figure 1 shows the flow diagram of the literature search. This systematic search identified 184 potential studies from MEDLINE journal (PubMed), Embase, and the Cochrane Library databases. 160 articles remained after excluding duplicate references. Of 160 studies, 143 studies were excluded after screening the titles and abstracts. After a careful review of the full texts of 17 articles, 10 articles were excluded because they did not fit the inclusion criteria. Among them, there is a included study (Oh, 2016) offered two cohort data: one is their original cohort performed in 2007–2011 to evaluate the tumor response to CRT associated with metformin use, recurrence-free survival, disease-free survival, and overall survival; the other is a validation cohort (2012–2014) to evaluate the positive metformin effect on pathological tumor response after CRT. Finally, we got eight cohorts from seven studies, with 2294 patients in total [9–15].

### Description of included studies

These seven cohort studies were published between 2012 and 2022. Three studies were conducted in Korea (Han 2022; Kim 2020; Oh 2016), two studies were conducted in the USA (Skinner 2013; Garrett 2012), and two studies were respectively carried out in Egypt (Shama 2021) and Spain (Planellas 2021). Furthermore, six studies reported the rate of pathologic complete response (pCR), four studies reported TRG, and three studies reported T/N downstaging rate. The male proportion in these studies ranged from 60 to 95%, and the mean age ranged from 52 to 65 years. The study included 506 patients with DM and 1788 patients without DM. The mean HbA1c levels in the DM group ranged from 5.7 to 7.7%. For the non-DM group, two cohorts and one cohort separately reported significantly lower BMI and HbA1c levels. Most of the included studies compared outcomes in three groups: DM + /MF + (with metformin use), DM + /MF − (without metformin use), and DM − /MF − . However, Shama (2021) (50 patients in total) and Garrett (2012) (38 patients in total) focused only on DM patients. The characteristics of the included studies were summarized in Table 1, which encompassed cohort time periods, diagnosis of DM and exposure to metformin, gender, age, BMI, HbA1c level, pretreatment CEA, response on tumor regression grade (TRG), and the proportion of pCR. Diagnosis criteria of diabetes patients, conducted metformin dosage, radiotherapy exposure dose, chemotherapy regimen, the operation details, and the staging system and response assessment of each study are listed in Supplementary file 3.

**Table 1:** Basic data and therapeutic effect of individual study

Author/year Country/included period	Patients^(DM+/MF+) (DM+/MF−) (DM−/MF−)^	Sex (male, %)	Age	BMI	HbA1c	Pre-CRT CEA	TRG (0–1)^c^, (%)	pCR (%)	Nos
			Mean ± SD, median [Q1–Q3], or median (range)						
Han (2021)	218^a^						73 (33.3)	36 (16.5)	9
Korea	30^DM+/MF−^	24 (80.0)	64.6 ± 8.7	23.6 [21.9–25.1]	6.7 [6.3–7.2]	3.5 [2.8–8.4]	**20 (66.7)**	10 (33.3)	
2010–2017	32^DM+/MF−^	30 (93.8)	61.8 ± 10.9	23.2 [20.9–25.9]	6.8 [5.8–7.9]	2.3 [1.9–3.8]	12 (37.5)	6 (18.8)	
	156^DM−/MF−^	114 (73.1)	62.1 ± 11.8	23.2 [21.2–25.0]	**5.3 [5.0–5.8]**	2.8 [1.9–4.1]	41 (26.7)	20 (12.8)	
Planellas (2021)	423		≥ 70 (%)	≥ 30 (%)					6
Spain	59^DM+/MF+^	**56 (94.9)**	29 (49.2)	18 (30.5)		3 [2–4]		**4 (6.8)**	
2010–2020	15^DM+/MF−^							4 (26.7)	
	349^DM−/MF−^	240 (65.9)^b^	134 (36.8)^b^	74 (20.3)^b^		3 [2–5]^b^		75 (21.5)	
Shama (2021)	50							14 (28.0)	8
Egypt	25^DM+/MF+^	21 (84.0)	58 (41–75)	32.4 (17–46)	5.7 (5–6.2)	1.9 (1.3–7.2)		**11 (44.0)**	
2018–2020	25^DM+/MF−^	15 (60.0)	52.7 (39–77)	28.3 (17–42)	5.9 (5.2–7.1)	1.7 (1.1–7.6)		3 (12.0)	
Kim (2020)	221					> 5 (%)	82 (37.1)	30 (13.6)	8
Korea	62^DM+/MF+^	64(77.4)	63.1 [60.6–65.6]	23.8 ± 3.3	7.4 (7.0–7.7)	17 (51.5)	**31 (50.0)**	**14 (22.3)**	
2000–2017	42^DM+/MF−^	27 (64.3)	63.4 [60.5–66.4]	23.8 ± 3.9	7.7 (7.2–8.2)	12 (44.4)	8 (19.0)	3 (7.1)	
	117^DM−/MF−^	80 (68.4)	60.2 [57.0–62.5]	23.3 ± 3.3	NA	43 (36.8)	43 (36.8)	13 (11.1)	
Oh (2016)	543						235 (43.2)	91 (19.3)	9
Korea	42^DM+/MF+^	27 (64.3)	62.5 (45–78)	25.0 (19.8–32.6)	6.9 (6.0–9.5)	3.4 (0.5–43.5)	**26 (61.9)**	11 (26.2)	
2007–2011	29^DM+/MF−^	24 (82.8)	65.0 (46–80)	24.1 (16.5–35.3)	6.7 (5.5–9.5)	3.4 (0.9–35.4)	10 (34.5)	6 (20.7)	
	472^DM−/MF−^	312 (66.1)	**54.0 (24–79)**	**23.6 (13.8–32.9)**	NA	2.6 (0.2–813.0)	199 (42.2)	74 (15.7)	
Oh’ (2016)	319						163 (51.1)	69 (21.6)	9
Korea	31^DM+/MF+^						**23 (74.2)**	6 (19.4)	
2012–2014	16^DM+/MF−^						7 (43.8)	2 (12.5)	
	272^DM−/MF−^						133 (48.9)	61 (22.4)	
Skinner (2013)	482								7
USA	20^DM+/MF+^	16 (80.0)	62 (43–74)	31 (22–48)	NA	2.7 (0.5–108)		**7 (35.0)**	
1996–2009	40^DM+/MF−^	29 (72.5)	63.4 (37–76)	31 (21–47)	NA	3.6 (0.5–30.2)		3 (7.5)	
	422^DM−/MF−^	263 (62.3)	**57 (19–84)**	**27 (17–70)**	NA	2.15 (0.4–185)		70 (16.6)	
Garrett (2012)	38								6
USA	19^DM+/MF+^						14 (73.7)		
2004–2008	19^DM+/MF−^						9 (47.4)		

*Abbreviations: BMI* body mass index, *HbA1c* glycated hemoglobin, *CRT* chemoradiotherapy, *CEA* carcinoembryonic antigen, *DM* diabetes mellitus, *MF* metformin, *NOS* Newcastle–Ottawa scale, *TRG* tumor regression grade, *pCR* pathological complete response (remission). The bold number means *p*-value < 0.05

^a^This extracted data excluded 14 patients with DM and started metformin treatment from CCRT initiated

^b^The basic data which was calculated with the 349^DM−/MF−^ group and 15^DM+/MF−^ group

^c^The TRG used in this study referenced the *AJCC Cancer Staging Manual*, 8th Edition. 0 means complete response (no remaining viable cancer cells), 1 means moderate response (only small clusters or single cancer cells remaining), 2 means minimal response (predominant fibrosis with residual cancer remaining), 3 means poor response (minimal or no tumor was killed or progressive cancer with extensive invasion)

**Fig. 2:**
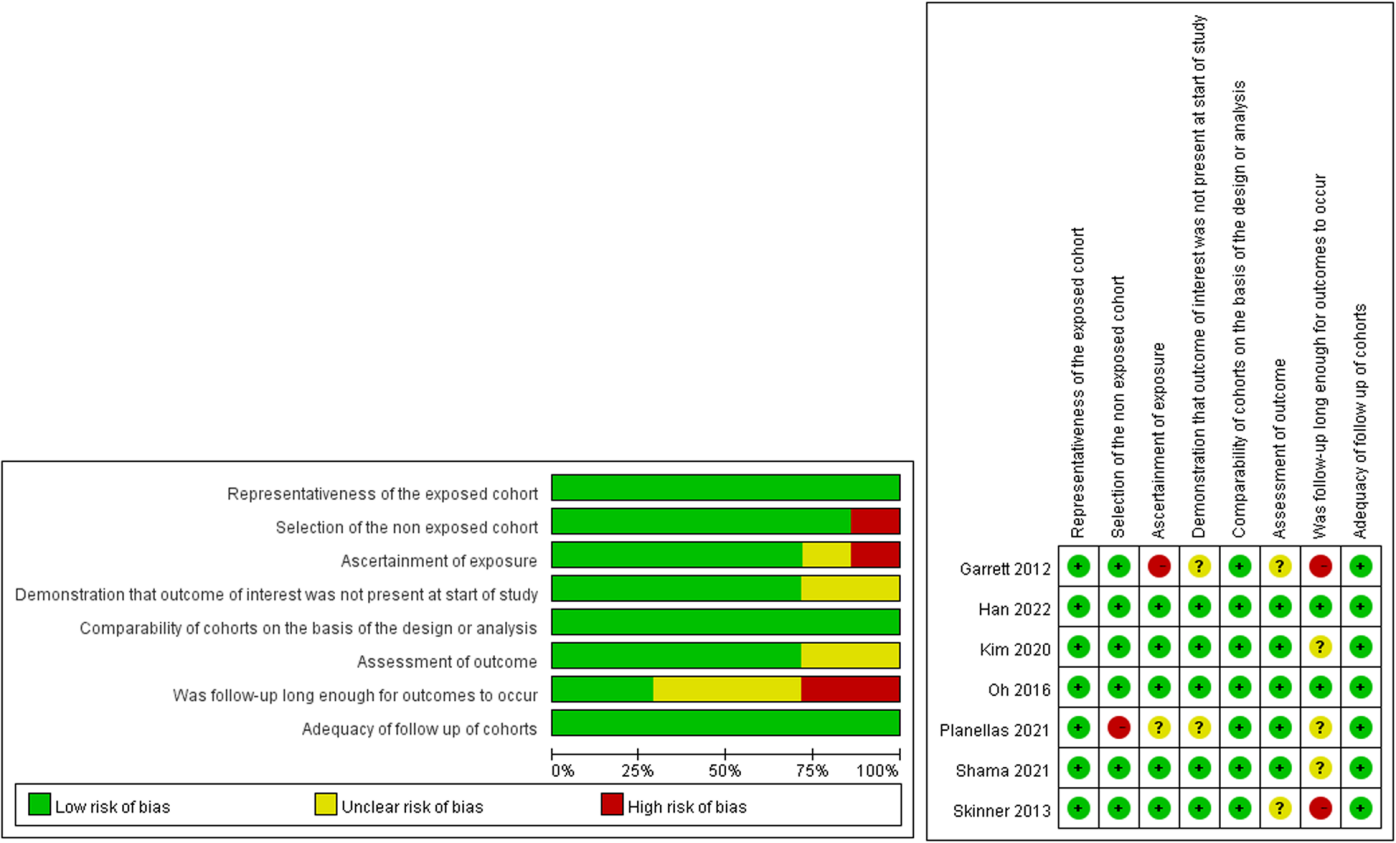
The summary of risk of bias in Newcastle–Ottawa scale. Green sign means low risk of bias, yellow sign means moderate risk of bias, and red sign means high risk of bias

The general qualities of the included evidence were optimal, except for two cohorts with NOS value of 6. The results are summarized in Fig. 2. For visually evaluating publication bias, we constructed funnel plots to assess the symmetry of pCR and TRG (Supplementary file 2).

### Pathological complete response

**Fig. 3:**
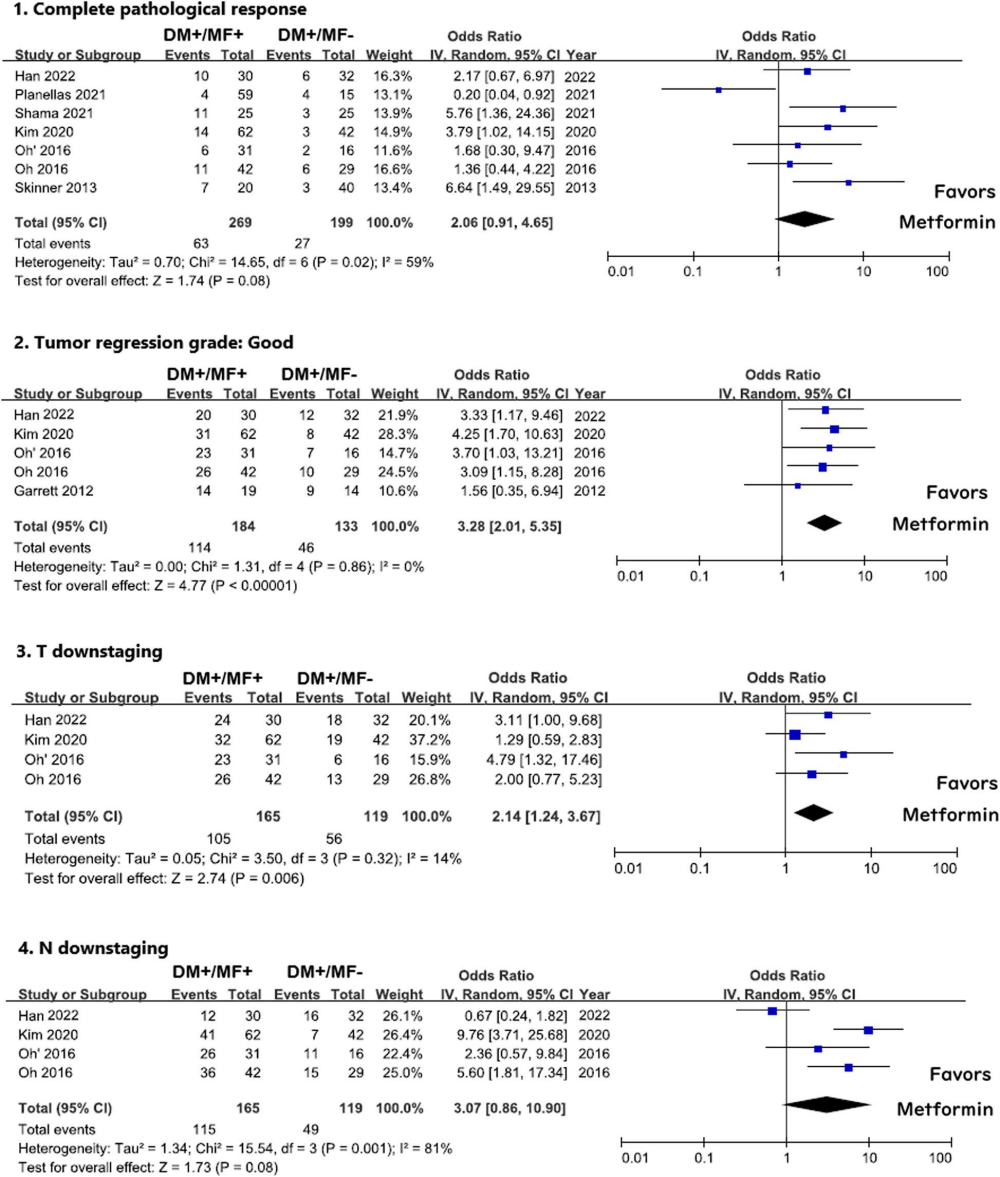
The summary of odds ratios of pCR, TRG, T downstaging, and N downstaging between DM patients with metformin (DM + /MF +) and DM patients without metformin (DM + /MF −). Oh (2016) has two cohorts: Oh 2016 (neoadjuvant CRT followed by surgical resection from 2007 to 2011) and Oh 2016’ (neoadjuvant CRT followed by surgical resection from 2012 to 2014)

**Fig. 4:**
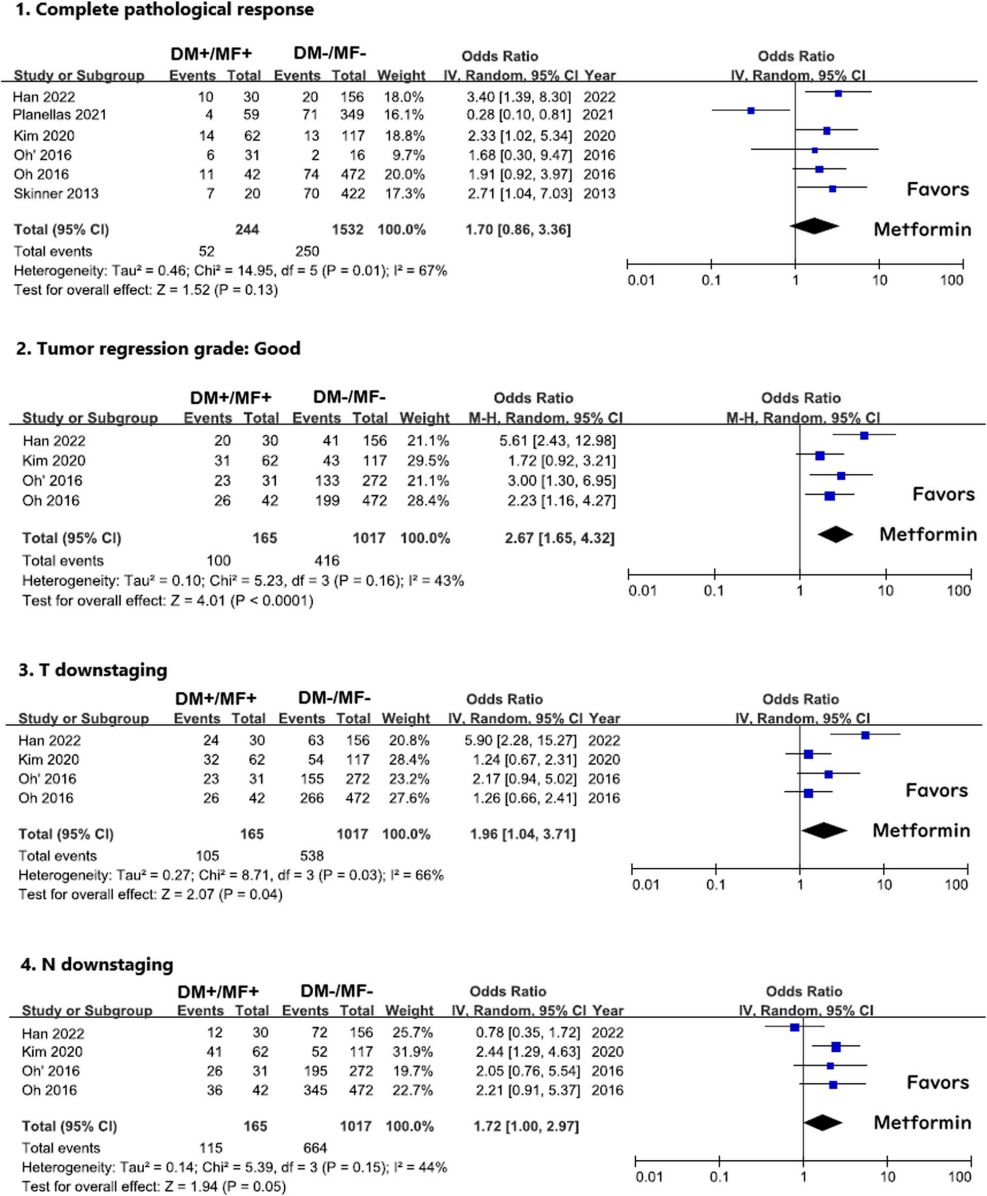
The summary of odds ratios of pCR, TRG, T downstaging, and N downstaging between DM patients with metformin (DM + /MF +) and non-DM patients (DM-/MF −). Oh (2016) has two cohorts: Oh 2016 (neoadjuvant CRT followed by surgical resection from 2007 to 2011) and Oh 2016’ (neoadjuvant CRT followed by surgical resection from 2012 to 2014)

There were seven cohorts that provided pCR of the removed pathological tissue of the rectal cancer patients [9, 10, 12–15]. In these studies, we got 269 DM cases using metformin (DM + /MF +), 199 DM cases using other antidiabetic agents (DM + /MF −), and 1532 nondiabetic cases (DM − /MF −). The pooled OR was 2.06 with CI from 0.91 to 4.65. (*I*^2^ = 59%, *p* = 0.08) for DM cases using metformin versus DM cases without using metformin (Fig. 3–1). For 244 DM patients treated with metformin versus 1532 nondiabetic patients, the pooled OR was 1.70 (*CI*: 0.86–3.36, *I*^2^ = 67%, *p* = 0.130) [9, 10, 12, 13, 15] (Fig. 4–1). The overall proportion of pCR was 23.4% in the DM patients using metformin, 13.6% in the DM patients without using metformin, and 16.3% in nondiabetic patients.

### Tumor regression grade (TRG)

In this study, we adopted a TRG system according to the American Joint Committee on Cancer (AJCC). TRG 0 and 1 in AJCC were regarded as “good response” to neoadjuvant CRT. Five cohorts provided TRG of the resected pathological tissue in diabetic rectal cancer patients [9, 11, 12, 15], which included 184 cases treated with metformin and 133 cases treated with other hypoglycemic agents. The pooled OR was 3.28 (*CI*: 2.01–5.35, *I*^2^ = 0%, *p* < 0.001) (Fig. 3–2).

Another comparison used 165 DM patients treated with metformin and 1017 nondiabetic cases [9, 12, 15] (Fig. 4–2). The pooled OR of these remaining 4 cohorts was 2.67 (*CI*: 1.65–4.32, *I*^2^ = 43%, *p* < 0.001).

Notably, in TRG comparison, we excluded one of the cohorts because this study comprised total 364 cases without metformin exposure, but 15 cases of them had a diagnosis of DM [13]. Besides, the study defined good response as *TRG* = 1 to 3 according to Mandard system, which was different from the other studies.

The overall proportion of “good response” to neoadjuvant CRT was 62.0% in the DM patients using metformin, 34.6% in the DM patients without metformin, and 40.9% in non-DM patients.

### T and N downstaging

Four cohorts provided T and N downstaging after neoadjuvant CRT treatment [9, 12, 15]. The pooled OR of T and N downstaging were 2.14 (*CI*: 1.24–3.67, *I*^2^ = 14%, *p* = 0.006) and 3.07 (*CI*: 0.86–10.90, *I*^2^ = 81%, *p* = 0.08) comparing metformin (*n* = 165) or non-metformin (*n* = 119) use in diabetic rectal cancer patients (Fig. 3–3, 4). Furthermore, when comparing with nondiabetic group (*n* = 1017), the pooled OR of T and N downstaging were 1.96 (*CI*: 1.04–3.71, *I*^2^ = 66%, *p* = 0.04) and 1.72 (*CI*: 1.00–2.97, *I*^2^ = 44%, *p* = 0.05) (Fig. 4–3, 4).

## Discussion

In this systematic review and meta-analysis, we aimed to pool the chemo-radio effect of metformin on advanced rectal cancers from clinical studies. We concluded better responses in TRG staging and T downstaging, slightly higher pCR rate, and better N downstaging in diabetic metformin users with rectal cancer by neoadjuvant CRT from 2294 patients.

The heterogeneity was contributed mainly by the article from Planellas et al. They concluded that taking metformin made the patients’ response worse to neoadjuvant CRT [13]. In the discussion of this study, the authors explained that the differences of the chemotherapy regimens, patient including times, and the intervals between neoadjuvant therapy and surgical resection may be the possible reasons why their conclusion was contrast to others. However, the details of how they confirm the diagnosis of diabetes, the duration or dose of metformin, and the HbA1c data were not provided in the article. In addition, Han et al. reported a contrary result of N downstaging; however, the authors did not explain why the results of their N downstaging were different to the others [15].

Studies have shown that diabetic patients have increased risks for breast cancer, pancreas cancer, uterine carcinoma, and CRCs [16]. The increased risk of malignancy in DM patients might be related to elevated circulating insulin level which results in cell growth and proliferation [17]. The substantial effect of type 2 DM, such as oxidative stress and chronic inflammation, also has an impact on the onset and progression of CRCs [18]. A large meta-analysis including 1,025,034 patients reported that with underline DM, the CRC patients had significantly declined overall survival and elevated all-cause mortality [19]. Another meta-analysis noted that in comparison to patients without DM, rectal cancer patients with DM would have a 16% shorter overall survival [20].

Patients with increased pCR rates were associated with better prognoses and could be eligible for less invasive surgeries or even a “watch-and-wait” approach that resulted in excellent rectal preservation [21–23]. On the other hand, patients with type 2 DM and CRC who receive curative surgery might have increased mortality rate and worse prognosis. Metabolic syndrome, which is a significant risk factor for type 2 DM, had an increased risk of 30-day postoperative complications in elective CRC surgeries [24]. For those patients with DM or multiple comorbidities, “watch and wait” might be a safer choice if the oncologic outcome is similar. Therefore, identifying modifiable factors that increase pathological responses after neoadjuvant CRT was valuable in rectal cancer patients.

Metformin, as one of the preferred first-line oral glucose-lowering agents, has growing evidence showing its anticancer effect in many ways [17, 25, 26]. In numerous studies, metformin has been reported to have a potential chemoprevention effect on colorectal polyps, colorectal adenoma, and/or rectal aberrant crypt foci [27, 28]. In patients with type 2 diabetes, metformin is associated with a lower incidence of colorectal cancer [29, 30]. Metformin was reported to reduce gastrointestinal radiotoxicity and could increase radiosensitivity of colorectal cancers [31]. Activation of AMP-activated protein kinase (AMPK) is demonstrated as one of antineoplastic effects of metformin [32].

### Mechanism of metformin on cancer cell apoptosis

**Fig. 5:**
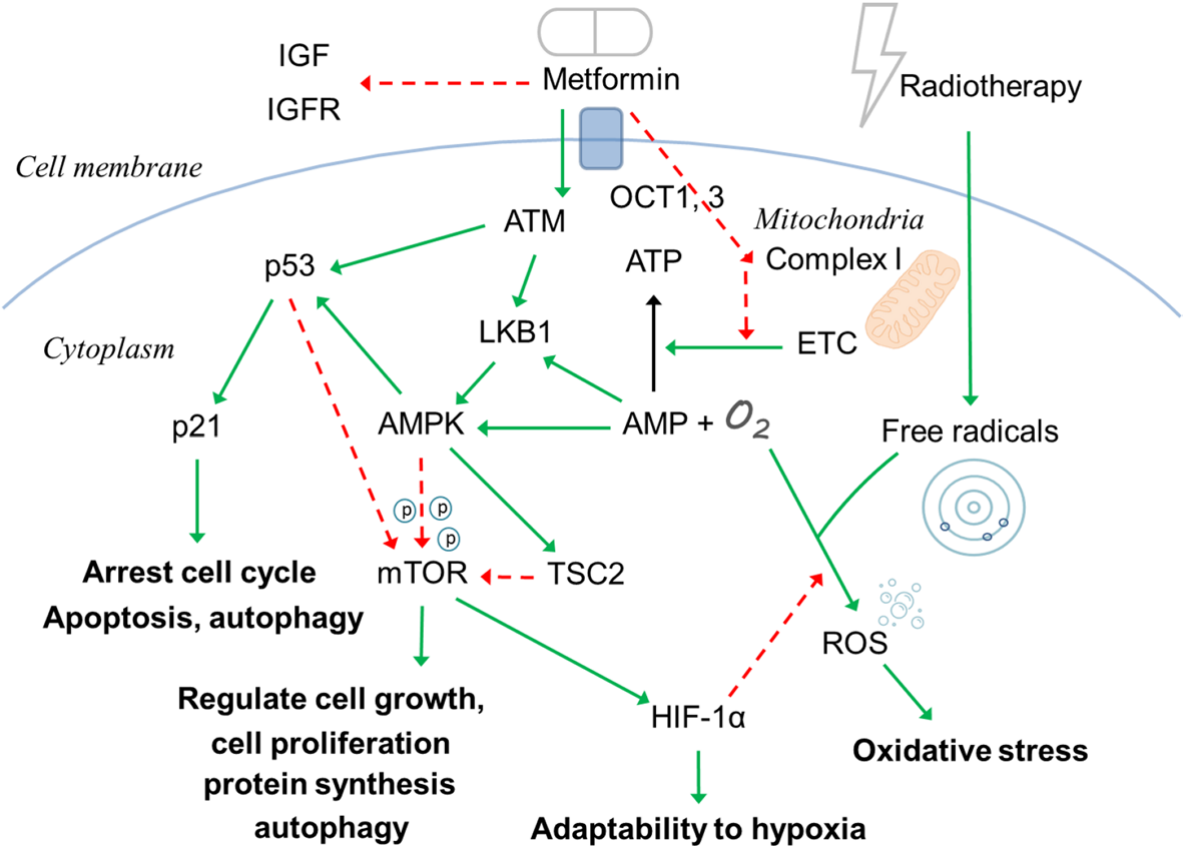
The summary of chemo-radio effect of metformin. After administration, the hypoglycemic effect of metformin decreases insulin level and inhibits insulin-like growth factor (IGF) signaling pathway as the indirect anticancer effect. In the direct anticancer pathways, metformin is transported by OCT-1 and OCT-3 to mitochondria, inhibits the ETC to generate ATP, and preserves oxygen. The ATM, LKB1, and AMPK pathway illustrate the anticancer effects on p53 and mTOR. The inhibition on mTOR to HIF-1α, free radicals generation, and ROS illustrate the regulatory effects of metformin on radio-refractory cancer cells. The green arrow means enhance or upregulate; the red arrow means inhibit or downregulate

The association of AMPK with the anticancer effect of metformin is essential. Organic cation transporters 1 and 3 on the cell membrane introduce metformin into the cell, and they affect complex 1 of the electron transfer chain (ETC) at the mitochondria [33]. This activity produces pathologic stress resulting in a decrease in adenosine triphosphate (ATP) and an increase in AMP. Liver kinase B1 (LKB1), a tumor suppressor, is activated by increasing AMP and acts as a sensor of cellular energy charges. The ataxia-telangiectasia-mutated (ATM) gene, which has an essential role in controlling and regulating the cellular cycle, is also a tumor suppressor that can phosphorylate LKB1 as a response to metformin. AMPK is the downstream component of LKB1, and it is activated and involved with the regulation of mammalian target of rapamycin (mTOR) activity, which frequently alters its signaling pathway in cancer. AMPK inhibits mTOR activity by phosphorylating co-signaling molecules and activating the tuberculous sclerosis complex 2 (TSC2). Phosphorylated co-signaling molecules are attached to mTOR, while phosphorylation of TSC2 directly inhibits the activity of mTOR. The inhibition of mTOR could stop cell growth and decrease cell proliferation [17, 25, 34] (Fig. 5).

As mentioned above, the inhibition of complex 1 on ETC by metformin reduces intracellular oxygen consumption and increases reactive oxygen species (ROS). ROS are associated with deoxyribonucleic acid (DNA) damage and decreased protein and lipid production. The activated AMPK by metformin leads to the expression of p53. p53 is a tumor suppressor gene that affects transcription of the p21 gene, another tumor suppressor gene. The activation of p53 and p21, which enhance cell autophagy and apoptosis, is initiated by metformin and radiotherapy, and their activation triggers a deceleration or end of the cell cycle [35] (Fig. 5).

### Mechanism of metformin on chemo-radioresistant cancer cells

Ionizing radiation induces DNA damage by generating free radicals and increasing ROS. Free radicals need oxygen to induce oxidative stress, which causes permanent DNA damage and cell death. Therefore, the development of tumor hypoxia and the following metabolic pathways is associated with clinical radio resistance [36]. With this microenvironment feature, tumors adapt to hypoxia partially by the activation and stabilization of hypoxic-inducible factors (HIFs). HIF transcription factors, which are composed of three isoforms (HIF-1α, HIF-2α, and HIF-3α), are involved with adaptation to hypoxia during radiotherapy. Hypoxia and HIF-1α increase glucose channeling into aerobic glycolysis, which is what most cancer cells prefer. This Warburg effect results in abnormal proliferation and malignant progression of cancer cells. HIF-1α requires mTOR to regulate the activity. As a result, AMPK indirectly inhibits HIF-1α activity and makes cancer cells fragile during radiotherapy [17, 37] (Fig. 5). By downregulation of HIF-1α activity, metformin could overcome hypoxic radioresistance through inhibition of mitochondrial respiration and induce autophagy and apoptosis in CRC cells [38, 39].

Our study is not devoid of limitations. First, the available studies included in this meta-analysis were still insufficient due to the low patient number of rectal cancers undergoing neoadjuvant CRT with diabetes and metformin usage. There is no randomized control study published to date. Second, it is the existence of variable heterogeneity and possible publication bias. Planellas et al. [13] reported an inverse result that metformin was not associated with better tumor response. It also raised a concern that a study may not be published if it did not conclude a good response and metformin. Third, different strategies and regimens of neoadjuvant CRT, for example, 5-FU combined oxaliplatin or not, long-course vs. short-course RT, or the uneven intervals between CRT and surgery, may contribute to a different outcome. Fourth, the suggestive dose of metformin was concluded. Three included studies report the precise metformin dosage used in their studies; however, the dosages were variable (details displayed in supplement 3). Also, Shama et al. [14] performed the subgroup analysis on 25 diabetic patients divided by the metformin dose they used. However, the pCR difference was not significant between the subgroups. Fifth, the TRG systems used in the included studies were not consistent, and the interobserver variability may exist within single study or among the included studies.

## Conclusion

In this systematic review and meta-analysis, metformin administration was associated with better responses in TRG staging and T downstaging in patients with rectal cancer and type 2 DM. Moreover, in comparison with nondiabetic patients, diabetic patients with metformin administration had a superior response to neoadjuvant CRT on TRG and T/N downstaging. Well-designed, high-quality randomized controlled trials are warranted to establish the treatment modality of metformin in neoadjuvant chemoradiotherapy on rectal cancer patients.

## Supplementary Information

**Additional file 1: Supplementary file 1.** Information of search strategy.

**Additional file 2: Supplementary file 2.** Funnel plots for DM + /MF + vs. DM + /MF- and DM + /MF + vs. DM-/MF- in pCR and TRG.

**Additional file 3: Supplementary file 3**. Treatment details of individual research.

**Additional file 4**. PRISMA 2020 checklist.

## Data Availability

The datasets are available from the corresponding author on reasonable request.

## Abbreviations

AJCC TNM: American Joint Committee on Cancer tumor-node metastasis
AMP: Adenosine monophosphate
AMPK: AMP-activated protein kinase
APR: Abdomino-perineal resection
ATM: Ataxia-telangiectasia mutated
ATP: Adenosine triphosphate
BMI: Body mass index
CCRT: Concurrent chemoradiotherapy
CEA: Carcinoembryonic antigen
CI: Confidence interval
CRC: Colorectal cancer
CRT: Chemoradiotherapy
DM: Diabetes mellitus
DNA: Deoxyribonucleic acid
ETC: Electron transfer chain
HbA1c: Glycated hemoglobin
HIFs: Hypoxic-inducible factors
LKB1: Liver kinase B1
MF: Metformin
mTOR: Mammalian target of rapamycin
NOS: Newcastle–Ottawa scale
OHA: Oral hypoglycemic agents
OR: Odds ratio
pCR: Pathological complete response (remission)
ROS: Reactive oxygen species
RT: Radiotherapy
TRG: Tumor regression grade
TSC2: Tuberculous sclerosis complex 2
5-FU/LV: 5-Fluorouracil/leucovorin
