# Supplementary material for: Metformin increases pathological responses to rectal cancers with neoadjuvant chemoradiotherapy: a systematic review and meta-analysis
Source: World J Surg Oncol. 2023 Jul 26;21:224. doi: 10.1186/s12957-023-03087-6 (PMC10369710; doi:10.1186/s12957-023-03087-6)
Supplement: Supplementary file 1 — Additional file 1: Supplementary file 1. Information of search strategy. [file 12957_2023_3087_MOESM1_ESM.docx]

**Pubmed (17): Search strategy and key words**

#1 (((((((((neoadjuvant chemoradiation therapy) OR (preoperative chemoradiation therapy)) OR (preoperative treatment)) OR (neoadjuvant treatment)) OR (neoadjuvant therapy)) OR (neoadjuvant chemoradi*)) OR (preoperative chemoradi*)) OR (neoadjuvant radiochemotherapy)) OR (preoperative radiochemotherapy)) OR (preoperative therapy)

#2 (((((rectal neoplasm) OR (rectal cancer)) OR (rectal adenocarcinoma)) OR (rectal carcinoma)) OR (rectal tumor)) OR (rectal tumour)

#3 (((metformin) OR (oral hypoglycemic agent)) OR (oral hypoglycemic medication)) OR (oral hypoglycemic drug)

#1 and #2 and #3: **17 results**

**Medline (126): Search strategy and key words**

#1 exp Colorectal neoplasms/

#2 CRC.tw.

#3 ((colon* or colorect* or rectal or rectum or bowel) adj (carcinom* or neoplas* or adenocarcinom* or cancer* or tumor* or tumour* or sarcom* or malignan* or metastat*)).tw.

#4 (radi* or chemoradi* or radiochemo* or chemo*).tw.

#5 exp metformin/

#6 metformin.tw.

#7 Biguanide*.tw.

#8 Hypoglycemic Agents/

#9 #1 or #2 or #3

#10 #5 or #6 or #7 or #8

#11 #9 and #10 and #4: **126 results**

**Embase (27): Search strategy and key words**
#1 colorectal AND neoplasms OR crc:ab,ti OR ((colon*:ab,ti OR colorect*:ab,ti OR rectal:ab,ti OR rectum:ab,ti OR bowel:ab,ti) AND adj:ab,ti AND (carcinom*:ab,ti OR neoplas*:ab,ti OR adenocarcinom*:ab,ti OR cancer*:ab,ti OR tumor*:ab,ti OR tumour*:ab,ti OR sarcom*:ab,ti OR malignan*:ab,ti OR metastat*:ab,ti))

#2 metformin OR biguanide*:ab,ti OR 'hypoglycemic agents':ab,ti OR 'hypoglycemic medication':ab,ti OR 'hypoglycemic drug':ab,ti

#3 radi* OR chemoradi* OR radiochemo* OR preoperative:ab,ti OR neoadjuvant:ab,ti

#4 #1 and #2 and #3: **27 results**

**Cocrane Library (14): Search strategy and key words**

#1 Title Abtract keyword: (colon* or colorect* or rectal or rectum or bowel or intestin*) AND (carcinom* or neoplas* or adenocarcinom* or cancer* or tumor* or tumour* or sarcom* or malignan* or metastat*)

#2 Title Abtract keyword: radi* or chemoradi* or radiochemo* or preoperative or neoadjuvant

#3 metformin or biguanide* or "hypoglycemic medication" or "hypoglycemic drug" or "hypoglycemic agent"

#4 #1 and #2 and #3: **14 trials**
