# Supplementary figures and images for: Metformin increases pathological responses to rectal cancers with neoadjuvant chemoradiotherapy: a systematic review and meta-analysis
Source: World J Surg Oncol. 2023 Jul 26;21:224. doi: 10.1186/s12957-023-03087-6 (PMC10369710; doi:10.1186/s12957-023-03087-6)

## 1. Funnel plot of pCR

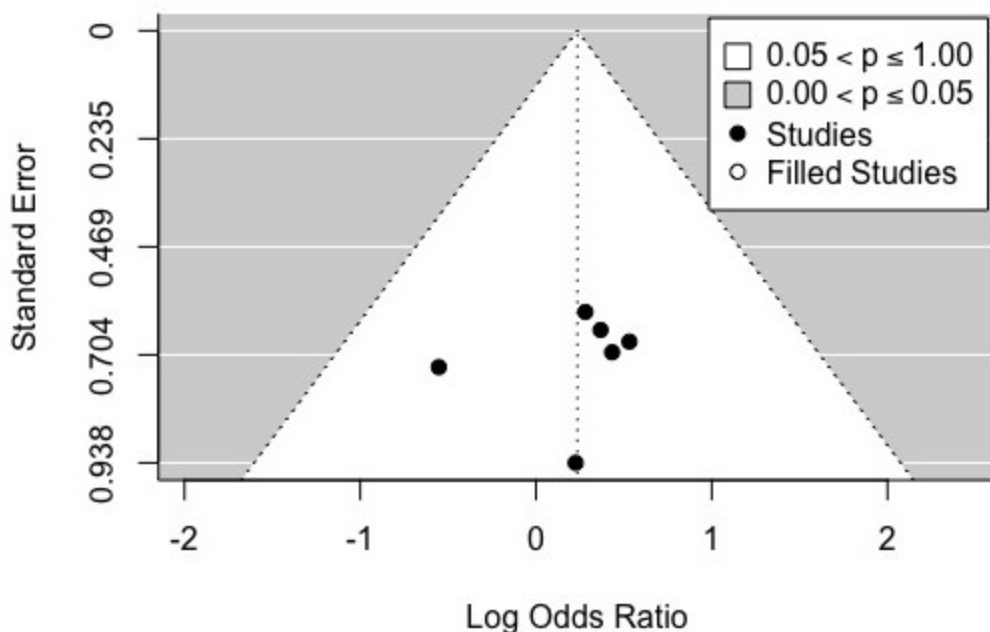

## 2. Funnel plot of TRG

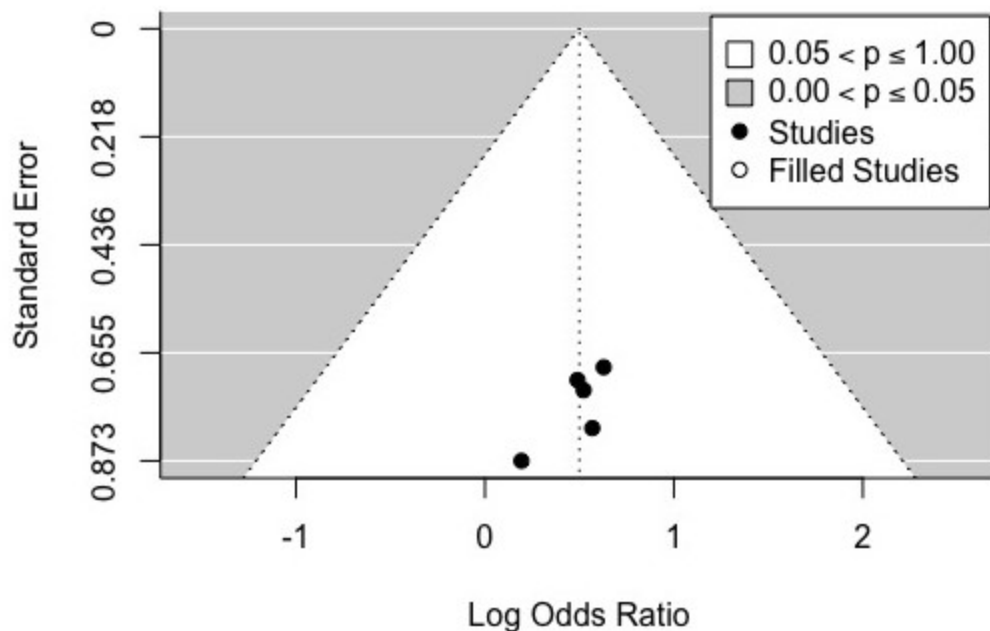

Supplement: Supplementary file 2 — Additional file 2: Supplementary file 2. Funnel plots for DM + /MF + vs. DM + /MF- and DM + /MF + vs. DM-/MF- in pCR and TRG. [file 12957_2023_3087_MOESM2_ESM.pdf]
