# Supplementary material for: Metformin increases pathological responses to rectal cancers with neoadjuvant chemoradiotherapy: a systematic review and meta-analysis
Source: World J Surg Oncol. 2023 Jul 26;21:224. doi: 10.1186/s12957-023-03087-6 (PMC10369710; doi:10.1186/s12957-023-03087-6)
Supplement: Supplementary file 3 — Additional file 3: Supplementary file 3. Treatment details of individual research. [file 12957_2023_3087_MOESM3_ESM.docx]

**Supplement 3** Therapeutic details of individual study

| Study | DM diagnosis criteria | Metformin dose and exposure periods | Radiotherapy dose | Chemotherapy | Operation | TRG staging system |
| --- | --- | --- | --- | --- | --- | --- |
| Han 2021 | Compliance with metformin was checked with continuous follow-up by an  endocrinologist. | Median: 1000mg daily [IQR = 250 – 4000mg] | The whole irradiation dose was 44-54 Gy, and 1.8-2.0 Gy was irradiated per day | 5-FU (infused for 5 days in the first and fifth weeks  of RT) or capecitabine (twice daily during  RT) | Total mesorectal excision (TME) was performed about 8 weeks after  CCRT | Mandard (1 – 2: good, 3 – 5: poor) |
| Planellas 2021 | N/A | N/A | Fractioned radiation (1.8 Gy/d; total dose, 45-50.4 Gy) | 5-FU (79.7%), capecitabine (13.9%) or combined the both (6.4%). | Median 10 weeks from the completion of CRT to surgery. | AJCC TNM staging system, Mandard (1 – 3: good, 4 and 5: poor) |
| Shama 2021 | Antidiabetic medications were identified through patient’s medical records. | 250 mg once to 850 mg three times daily | 1.8–2.0 Gy daily fractions for a total dose of 54 Gy | 5-FU (36%) (infused for 5 days in the first and fifth weeks  of RT) or capecitabine (64%) (twice daily during  RT) | Surgery resection (82% APR) between 6 and 12 weeks (median: 8 weeks) after the completion of neoadjuvant CCRT. | AJCC TNM staging system |
| Kim 2020 | Diagnosed with DM by independent experts and were receiving OHAs | use MF > 6 months before cancer diagnosis | Pelvic radiotherapy for 5 weeks, 49–51 Gy in total | 5-FU/LV (60%) or capecitabine (19%) | Curative surgical resection at approximately 8 weeks after completion of neoadjuvant CCRT | AJCC TNM staging system |
| Oh 2016  Oh’ 2016 | Criteria of American Diabetes Association | 1000mg daily (range: 250 – 2550mg) | 1.8–2.0 Gy daily fractions, 44–54 Gy in total | 5-FU/LV (66%) or capecitabine (32%) | Total mesorectal excision between 6 and 10 weeks after the completion of neoadjuvant CCRT | Dworak’s (0 – 2: poor, 3 and 4: good) |
| Skinner 2013 | Preexistence or a new diagnosis of diabetes prior to chemoradiation | Being taken MF by the patient at the start of therapy | 50.4 (20–63, range) Gy in total | 5-FU (53%) or capecitabine (45%) | Proctectomy with coloanal  anastomosis, low anterior resection, or abdominoperineal  resection | pCR was defined as an absence of tumor cells in the primary specimen and sampled lymph nodes. |
| Garrett 2012 | By review of outside records as having type II DM; additional  confirmatory testing was not routinely performed. | Treated with MF as one of their diabetic medications | N/A | N/A | N/A | complete and minor pathologic response rate (< 10% residual tumour) |

*Abbreviations*: AJCC TNM: American Joint Committee on Cancer tumor-node-metastasis, APR: Abdomino-perineal resection, CCRT: Concurrent chemoradiotherapy, DM: Diabetes mellitus, MF: Metformin, OHA: Oral hypoglycemic agents, RT: radiotherapy, TRG: tumor regression grade, 5-FU/LV: 5-fluorouracil/leucovorin.
